# Tannic acid inhibits TNF-α signaling by targeting the protein disulfide isomerase and alleviates symptoms in an imiquimod-induced psoriasis mouse model

**DOI:** 10.1186/s12964-025-02535-y

**Published:** 2025-11-29

**Authors:** Wenhua Jin, Yi Xia, Shuo Sun, Hejing Tang, Senyang Hu, Yan Zhang, Jiaqiang Huang, Ping Liu, Chenyun Hu, Jiayue Guo, Pengjie Wang, Peng An, Junjie Luo, Lei Wang, Fuqing Wang, Yongting Luo, Yinhua Zhu

**Affiliations:** 1 Department of Nutrition and Health, Beijing Advanced Innovation Center for Food Nutrition and Human Health, China Agricultural University, Beijing, 100193 China; 2 State Key Laboratory of Biomacromolecules, Institute of Biophysics, Chinese Academy of Sciences, Beijing, 100101 China; 3 College of Food Science and Engineering, Gansu Agricultural University, Lanzhou, 730070 China; 4 Tibet Tianhong Science and Technology Co., Ltd, Xizang, 850000 China

**Keywords:** Tannic acid, PDI inhibitor, TNF-α signaling, TNFR1 shedding, Psoriasis

## Abstract

**Background:**

Inhibiting TNF-α signaling is an effective approach to prevent inflammation, which can mitigate the symptoms of autoimmune diseases. Activation of the ADAM17-TNFR1 signaling module using small-molecule protein disulfide isomerase (PDI) inhibitors effectively induces TNFR1 shedding and TNF-α signaling inhibition. However, it is not known whether tannic acid (TA), a verified PDI inhibitor with outstanding anti-inflammatory effects, could alleviate autoimmune diseases.

**Objective:**

We set out to explore the anti-inflammatory mechanism of TA and whether it could be used to treat the classical autoimmune disease, psoriasis.

**Methods:**

Molecular interactions were assessed using insulin reduction assays with full-length PDI and its domain fragments to identify TA binding sites. Non-covalent binding and conformational changes were evaluated using AMS-modified SDS-PAGE and ANS fluorescence. Molecular chaperone activity was measured using rhodanese refolding. Cellular assays included cytotoxicity, apoptosis, and NF-κB activation in L929 cells using CCK-8, flow cytometry, western blot, and RT-qPCR. PDI dependency was confirmed using CRISPR-Cas9 knockout. TNFR1 shedding was quantified using flow cytometry and ELISA. In vivo efficacy was tested in an imiquimod (IMQ)-induced psoriasis mouse model treated with TA ointment (5% and 10%), and the outcomes were evaluated using the psoriasis area and severity index (PASI), histopathology, blood routines, and blood biochemical examinations.

**Results:**

TA selectively inhibited the reductase activity of the **b’** domain of PDI and induced non-covalent conformational changes, reducing hydrophobicity and chaperone function. TA effectively suppressed TNF-α-induced apoptosis in cells, NF-κB activation, and inflammatory gene expression. PDI knockout abolished TA-induced TNFR1 shedding, confirming PDI dependence. In IMQ-induced psoriatic mice, 10% TA ointment significantly reduced the PASI scores and the incidence of histopathological features. TA also normalized blood inflammation and restored physical functions.

**Conclusions:**

In summary, our study showed that TA blocks TNF-α signaling by inhibiting PDI, and exhibits potential application value in combating autoimmune diseases, especially psoriasis.

**Supplementary Information:**

The online version contains supplementary material available at 10.1186/s12964-025-02535-y.

## Introduction

Psoriasis is a common chronic inflammatory skin disease. More than 125 million people worldwide suffer from it, and its prevalence rate is increasing annually [1]. Psoriasis patients experience symptoms such as papulosquamous plaques and erythema that are difficult to treat and they may also suffer from low self-esteem anxiety affecting their normal social interactions [2–4]. The pathogenesis of psoriasis and other autoimmune diseases is complex and involves epigenetic and environmental triggers, as well as immune function dysregulation [2]. These are closely related to many cytokine and signaling pathway regulators, such as the *IL12B* and *IL23R* genes [5], the CC chemokine CCL20 and its receptor CCR6 [6], and the interleukin-23/T-helper 17 Axis [7]. Tumor necrosis factor alpha (TNF-α) signaling acts as a key regulator of inflammatory responses, and it is instrumental in the pathogenesis of psoriasis [8, 9]. TNF-α is located as a trimer upstream of the TNF-α signaling pathway and plays key roles in autoimmune diseases by binding to either the 55 kDa cell membrane receptor TNFR1 or the 75 kDa cell membrane receptor TNFR-2 [10, 11]. Therefore, blocking the TNF-α signaling pathway is effective in the treatment of psoriasis and other autoimmune diseases.

Protein disulfide isomerase (PDI) is a crucial catalyst for protein folding in the endoplasmic reticulum of eukaryotic cells. It consists of four thioredoxin-like domains (**a**, **b**, **b’**, and **a’**) arranged in sequence from the N-terminal, with a C-terminal acidic extension c and an x-connector between the **b’** and **a’ **domains [12]. The redox-active Cys-Gly-His-Cys motifs of domains **a** and **a’** face each other across the central cleft and are responsible for thiol-disulfide interchange reactions [13], while the **b’** domain provides the main substrate binding site [12]. The U-shaped arrangement of these domains creates a hydrophobic surface within the cleft that enables specific binding to unfolded or misfolded substrates [13]. PDI plays a role in catalyzing the oxidation, reduction, and isomerization of disulfide bonds, and also functions as an active molecular chaperone [14]. It contributes to oxidoreductase, isomerase, and molecular chaperone activities during protein modification and folding [12]. It also participates in tissue factor decryption by catalyzing the exchange of a thiol-disulfide bond [15]. Reactions catalyzed by PDI play important roles in the pathogenesis of thrombosis [16, 17], tumors [18], neurodegenerative diseases [19], and aging [20]. Studies on the effect of PDI on TNF-α signaling regulation have been undertaken in recent years. It has been reported that PDI can directly interact with TNFRs, and that the PDI-inhibitor bacitracin can disrupt TNF/TNFR signaling [21]. Therefore, developing PDI inhibitors with the potential ability to block TNF-α signaling would contribute to the treatment of autoimmune diseases such as psoriasis.

We recently reported that the well-known natural product (−)-vinigrol can directly target and inhibit PDI, resulting in the activation of the protease ADAM17 as well as the shedding of TNFR1 from the plasma membrane, thus blocking TNF-α signaling [22]. Although vinigrol exhibits promising biological activity in inhibiting PDI and TNF-α signaling, it is in short supply due to its unique structure, thus limiting its widespread application [22]. It is therefore necessary to find effective and easily available vinigrol substitutes to use as a PDI inhibitor in the treatment of autoimmune diseases. The reported potent PDI inhibitors such as 16F16 and LOC14 have exhibited significant cytotoxic effects, raising concerns about their safety [23, 24]. Rutin, a flavanol glycoside found in buckwheat and tobacco leaves, *Ruta graveolens* leaves, and citrus fruit, is a well-understood PDI inhibitor which binds to the **b’ **domain of PDI [25]. However, rutin has been shown to be a less effective inhibitor of PDI than vinigrol at the cellular level [22]. Notably, its pharmacokinetic profile is reduced by its inherent instability and poor aqueous solubility, resulting in low systemic bioavailability [26, 27]. Consequently, further exploration of more easily available, effective, and safer PDI inhibitors is needed. Tannic Acid (TA) is a naturally occurring organic compound, found in a wide variety of common foods such as tea, coffee, beans, and unripe fruits [28]. Researchers have used molecular docking techniques to show that TA can act as a natural PDI inhibitor [29]. Furthermore, previous studies have shown the broad anti-inflammatory potential of TA, including its efficacy in allergic inflammatory skin disorders [30], supporting its relevance for inflammation-related disease research. However, it remains unknown whether TA exerts its effects by modulating the PDI-mediated TNF-α signaling pathway and whether this mechanism can be harnessed to alleviate autoimmune diseases such as psoriasis.

Here, we verify that TA binds non-covalently to the PDI protein, causing conformational changes that induce TNFR1 shedding, which results in effective inhibition of TNF-α signaling. The psoriasis symptoms in a mouse model were alleviated following TA treatment, restoring balanced physical function. Overall, our study showed that TA was able to block TNF-α signaling by inhibiting PDI, and exhibited potential application value in combating autoimmune diseases, especially psoriasis.

## Materials and methods

### Cytotoxicity measurement

L929 cells were cultured in Dulbecco-modified Eagle Medium (DMEM, Gibco Cell Culture Solutions, ThermoFisher, Waltham, MA, USA), 10% fetal bovine serum (FBS, Gibco Cell Culture Solutions, ThermoFisher, Waltham, MA, USA), and 1% penicillin-streptomycin (Beyotime, Shanghai, China) for cytotoxicity measurement. The experiment was carried out in 96-well plates (Corning Costar, New York, NY, USA). Each well was filled with 100 µL of culture medium with 1 × 10^5^ cells/mL and the plates were incubated overnight in a 37℃/5% CO_2_ cell incubator. The treated culture samples were dissolved in dimethyl sulfoxide (DMSO), then diluted in medium to the required concentrations and added to the plate wells. Healthy cells treated with DMSO formed the positive control. The treated cells were cultured for 8 h, then 20 µL MTS assay (Promega, Madison, WI, USA) solution was added to each well and incubated for 2 h at 37℃. Finally, the optical density (OD) of the medium was measured at 490 nm using a microplate spectrophotometer (iMark, Bio-Rad, Hercules, CA, USA). The blank control contained no cells, but only MTS solution. The cytotoxicity was calculated as follows: (OD_DMSO_-OD_compounds_)/(OD_DMSO_-OD_blank_) *100%. Data analysis was performed using GraphPad Prism.

### Assay for the Inhibition of TNF-α-induced L929 cytotoxicity

L929 cells were placed into 96-well plates with 1 × 10^4^ cells in each well. The plates were incubated overnight at 37℃ and 5% CO_2_. 10 ng/mL TNF-α and 1 µg/mL actinomycin D (Act D, Sigma-Aldrich, St Louis. MO, USA) were added to the cells for 8 h as a pretreatment to induce cell death. 50 µM and 100 µM TA (CAS No.: 1401-55-4, molecular formula: C_76_H_52_O_46_; molecular structure formula as shown in Fig. S1A; molecular weight 1701.20 g/mol; purity: HPLC ≥ 98%; Beite Renkang, Beijing, China) were added to the TNFα-induced cells for 8 h to inhibit cytotoxicity. 20 µM vinigrol was used as a positive control to decrease the TNF-α-induced cell death. The control was treated with TNF-α only. After 8 h of treatment, the culture medium was removed, and PBS was added to wash off the remaining reagents, after which a fresh 100 µL of culture medium was placed in each well and 20 µL MTS was added. The OD of the medium was determined using a spectrophotometer at 490 nm. The formula used to calculate the inhibitory effect of the chemicals on the TNF-α-induced cytotoxicity was: (OD_TNF−α + ActD + chemicals_-OD_TNF−α +ActD_)/(OD_ActD_ -OD_TNF−α + ActD_) * 100%. The data were analyzed using GraphPad Prism.

### Detection of the concentration of soluble TNFR1 using ELISA

L929 cells were cultured in a six-well plate, replaced with serum-free medium and stimulated with TA and vinigrol as described above. Then, cells were collected for flow cytometry and the culture medium was collected for ELISA assay. The concentration of soluble TNFR1 was detected using an ELISA kit (Elabscience, Wuhan, China). A neutravidin coated high-capacity plate was acclimated to room temperature and 275 µL PBS superblock was added to each well. The plate was incubated for 1 h at room temperature and then washed five times with PBST buffer (PBS, 0.01% Tween). Next, biotinylated TNFR1 protein, was dissolved in 1 nM PBS superblock and 100 µL of the solution was added to each well. The plate was agitated for 1 h on a thermomixer (Eppendorf, Hamburg, Germany) at 300 rpm. The plate was then washed five times with PBST buffer. The blank control consisted of PBS superblock without any added TNFR1. HRP-tagged TNFR1 protein was obtained by protein-HRP conjugation and dissolved with PBS superblock to 1 nM. The TNFR1 solution worked to dilute the TA and vinigrol culture medium. After dilution, 100 µL of solution was added to each well in the plate and incubated for 1 h at 37℃. The control was treated with TNFR1 solution mixed with DMSO. The plate was washed five times with PBST buffer. 100 µL of TMB solution (Elabscience, Wuhan, China), was added to each well and mixed thoroughly. The reaction was stopped by the addition of 50 µL of ELISA stop solution (Solarbio, Beijing, China) to each well. The OD of the solutions was measured at 450 nm using a spectrophotometer.

### Western blot analysis

Western blot analysis was used to detect whether TA could block TNF-α-induced NF-κB activation in the L929 cells. About 5 × 10^5^ L929 cells were plated onto six-well plates and incubated overnight at 37℃. Different combinations of drugs were diluted with DMEM containing 10% FBS as follows: DMSO; 10 ng/mL TNF-α + 1 µg/mL Act D; 10 ng/mL TNF-α + 1 µg/mL Act D + 20 µM vinigrol; and 10 ng/mL TNF-α + 1 µg/mL Act D + 100 µM TA. In order to detect the inhibitory effect of TA on TNF-α-induced NF-κB activation in L929 cells, the cells were pre-incubated for 4 h with DMSO, vinigrol, and TA, and then treated with TNF-α and Act D for 30 min before collection. The cells were washed twice with pre-cooled PBS buffer before harvesting. The total protein was extracted using a buffer (PBS, 0.5% Tween 20; Sigma Aldrich, St. Louis, MO, USA), and the protein concentration was normalized using a BCA protein assay kit (Beyotime, Shanghai, China). The total protein was mixed with 6×protein loading buffer, incubated at 95℃ for 5 min, and then about 50 µg protein was loaded per lane for western blot analysis. Western blot was also used to detect whether PDI knockout (KO) cells were effective. PDI KO L929 cells and L929 cells were cultured in a six-well plate until the wells were full. The antibodies used were as follows: NF-κB p65 antibody (Cat. #8242, Cell Signaling Technology, Danvers, MA, USA); NF-κB p50 antibody (Cat. #66992-1-Ig, Proteintech, Rosemont, IL, USA); IκBα Antibody (Cat. #9242, Cell Signaling Technology, Danvers, MA, USA); Anti-PDIA6 antibody (ab227545, Abcam, Cambridge, UK); Anti-β-Actin antibody, (Cat. #4970, Cell Signaling Technology, Danvers, MA, USA).

### Flow cytometry analysis

To detect the effect of TA on TNFR1 expression on the cell membrane, L929 cells were cultured on a six-well plate and treated with 50/100 µM TA, DMSO (control), and vinigrol (positive control) for 8 h. The cells were then harvested and resuspended in 100 µL PBS. The solution was marked with PE labeled anti-mouse CD120a (TNFR Type I/p55) (Cat. #113003, Biolegend, San Diego, California, USA) at a concentration of 1:200 and kept on ice for 30 min before washing with PBS solution. Fluorescent signaling analysis was performed with excitation at 488 nm and emission at 532 nm.

Cell apoptosis was also measured using flow cytometry. TNF-α and ActD were added to L929 cells for 8 h to induce cell apoptosis. Then TA, DMSO (control), and vinigrol (positive control) were used for 8 h to stimulate the apoptosis of L929 cells. Cells at the bottom of the dish were collected along with the cells in the medium. These cells were washed with PBS and resuspended in 195 µL Annexin binding buffer (Beyotime, Shanghai, China) and 5 µL Annexin-V-FITC (Beyotime, Shanghai, China). The cell suspension was incubated for 5 min at room temperature in the dark. After that, 10 µL PI (Beyotime, Shanghai, China) was mixed with the cell suspension and incubated for another 15 min. The results were analyzed using the FlowJo software.

### Knockout of PDI in L929 cells

The PDI KO cell line was constructed using CRISPR/Cas9 genome editing. The exon1 sequence of mouse PDI was targeted using guide RNA and cloned to the expression vector pSpCas9 (BB)−2 A-GFP (PX458) (Addgene https://www.addgene.org/, MA, USA). Single-guide RNA (sgRNA) was built to transfect L929 cells for 48 h using jet PRIME (Polyplus, Strasbourg, France), sgRNA1: TAGGGTGGGCGCCGACGCCC, sgRNA2: GAGCAACTTCGAGGAGGCGC. Then a FACS Aria-II sorter (BD BioSciences, Franklin Lakes, NJ, USA) was used to select GFP-positive cells and place them into a 96-well plate, one cell per well. The efficiency of the PDI KO was examined using western blotting.

### PDI related biochemical assay

Insulin reduction assay was used to measure the effect of TA on PDI activity. In the assay, 0.5 µM PDI (Sino Biological, Beijing, China) samples were incubated with either 0.8 µM TA, 3.6 µM rutin (positive control) (Selleck, Houston, TX, USA), or DMSO for 15 min at 37℃. Then, the suspension was mixed with 130 µM insulin (Sigma-Aldrich, St. Louis, MO, USA), 0.1 mM potassium phosphate buffer, pH 7.5, 2.5 mM EDTA, and 0.1 mM dithiothreitol (DTT). An EnSpire microplate reader (PerkinElmer, Waltham, MA, USA) was used to detect the absorbance of the suspension at 650 nm. Different concentrations of TA were incubated with PDI to measure the IC_50_ of the TA. The results were matched to a nonlinear curve. To detect the binding site of TA to PDI, PDI and PDI fragments (**a**, **ab**, **abb’**, **abb’x**,** bb’xa’**, **bb’xa’c**, **b’xa’c**, and **a’c**) were incubated with TA and rutin, following the protocols described above. The expression and purification of the PDI and PDI fragments were performed as described previously [31]. To obtain the ANS fluorescence spectra, 5 µM samples of PDI were treated with either 100 µM TA, DMSO (control) or 100 µM rutin (positive control) for 15 min at 37°C. 50 µM ANS samples were incubated with or without PDI in 50 mM Tris–HCl, 150 mM NaCl, pH 7.6 for 20 min at 25 °C in the dark. ANS fluorescence emission spectra were measured at 400–650 nm with excitation at 370 nm. The concentration of ANS was determined using an extinction coefficient at 350 nm and 5,000 M^− 1^cm^− 1^. The enhancement factor = [F (protein + ANS + buffer) – F (buffer)]/[F (ANS + buffer) – F (buffer)]: where F is the fluorescence intensity at 480 nm. Fluorescence spectra were recorded using an EnSpire Multimode Plate Reader (PerkinElmer, Waltham, MA, USA).

The reduced PDI fragments (**a**, **a’c**, **b’xa’c**) were prepared using DTT reduction as described previously [32]. The covalent binding ability of small molecule drugs to PDI fragments was confirmed by incubating 25 µM reduced PDI fragments (**a**, **a’c**, **b’xa’c**) with either DMSO, 2 mM AMS, 500 µM PACMA31, 500 µM rutin or 500 µM TA in 50 mM Tris-HCl, 150 mM NaCl, pH 7.0 for 20 min at room temperature in the dark. Then, 15% non-reducing SDS-PAGE was used to detect the electrophoretic migration behavior of PDI inhibitors after binding with PDI truncations.

In the chaperone activity assay, 1 µM PDI was mixed with 10 µM TA or DMSO for 2 h at 25℃. Then, 0.45 µM denatured rhodanese (Sigma-Aldrich, St. Louis, MO, USA) was added to the mixture and adjusted with 200 mM sodium phosphate, pH 7.5. Light scattering was monitored at 350 nm using an RF-5301PC Spectro-fluorophotometer (Shimadzu, Kyoto, Japan).

### Reverse transcription quantitative PCR (RT-PCR)

To indicate the inhibitory effect of TA on TNF-α-induced stimulation in L929 cells, we measured the expression of the related genes in the TNF-α pathway. L929 cells were treated with 100 µM TA or DMSO for 4 h. 10 ng/mL of TNF-α was then added to the cells for 0.5 h before RNA extraction. Reverse transcription and Accurate Biology RT-PCR (Accurate Biology, Hunan, China) were performed and the expression levels of *Il-1α*, *Mip2*, *Kcf*, and *Irf1* were calculated using GraphPad Prism.

### Experimental animals

Seven-week-old female BALB/c mice were acquired from the Beijing Vital River Laboratory Animal Technology Co., Ltd. and were acclimated to a 12 h/12 h light/dark environment at controlled temperature and humidity for seven days. The mice (body weight 16–20 g) were randomly divided into four groups (*n* = 6 in each group) according to body weight and treated as follows: (1) control (Vaseline ointment), (2) imiquimod model (IMQ), (3) 5% TA (IMQ + 5% TA ointment), (4) 10% TA ointment (IMQ + 10% TA ointment). The 5% TA ointment comprised: 4% TA, 4% glycerol, 72% Vaseline. The 10% TA ointment comprised: 8% TA, 6% glycerol, 66% Vaseline. Before the experiment, a 2 cm × 2 cm patch was shaved from the back hair of all of the mice. From day 0 to day 7, IMQ (Aldara, 3 M Health Care Ltd, Loughborough, UK) was applied to the back skin of every mouse at the rate of 62.5 mg/kg of body weight, except those in the blank control which were treated with Vaseline in the morning. 5% or 10% TA was applied to the IMQ-treated skin in the afternoon, except for the control and IMQ groups treated with Vaseline. The back skin of the mice was photographed. The rash, scurf area, and severity index of all mice were recorded from day 0 to day 7. The animals were sacrificed after CO_2_ anesthesia on Day 7 and blood and skin tissue samples were collected. The animal research protocol was approved by the Experimental Animal Ethics Committee of the China Agricultural University (approval number: AW40704202-5-1, dated 2024.07.04).

### Hematoxylin and Eosin (HE) staining

The back skin of the mice was dehydrated and embedded in paraffin. The samples were dyed using a HE staining kit (Beyotime, Shanghai, China). Pathological features, including hyperkeratosis with hypokeratosis, acanthosis thickening, thinning or absence of the granular layer, and so on, were scored according to the severity of the pathology.

### Statistical analysis

GraphPad Prism 8 (GraphPad Software Inc.; San Diego, CA, USA) was used to calculate the *P* values of t-tests (to compare the differences between two groups) and for one-way ANOVA (to compare more than two groups), or two-way ANOVA (to determine statistical differences between groups in vivo), with default parameters. Data are presented as means ± SEM, with n as the number of experiment replicates or the number of mice. *P* values are shown as follows: *P < 0.05; **P < 0.01; and ***P < 0.001. The results were obtained from at least three independent experiments for the in vitro experiments. Animal experiments were conducted with *n* = 6 per group for the in vivo therapeutic evaluations.

## Results

### TA inhibits PDI by binding to the b’ domain in an insulin reduction assay

**Fig. 1:**
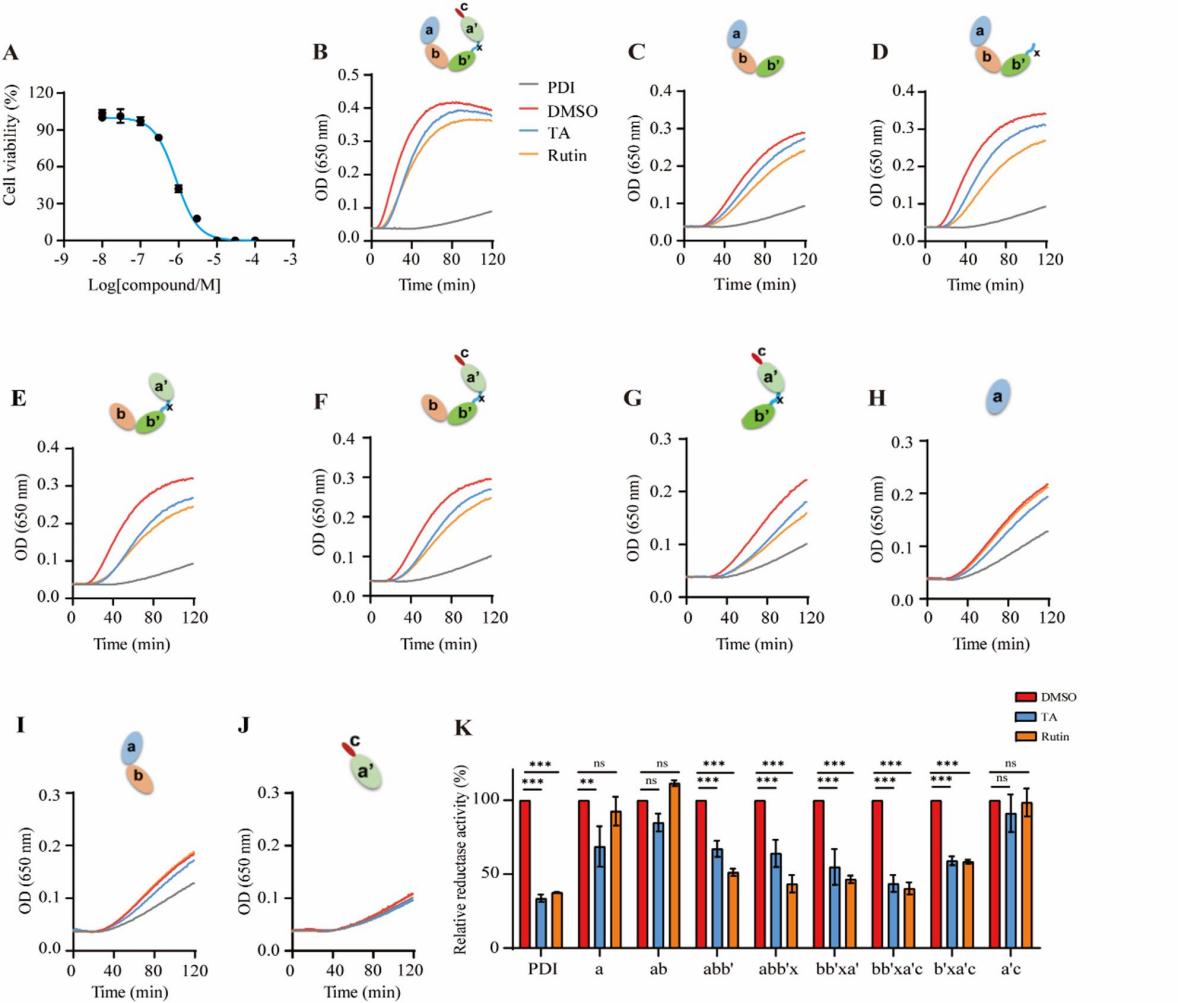
Tannic acid (TA) inhibits protein disulfide isomerase (PDI) by binding to the b’ domain in insulin reduction assays.** A**. IC_50_ of TA on PDI inhibition, as detected by insulin reduction assay. **B–J**. The inhibitory effect of TA on PDI and PDI fragments, as evaluated using either full-length PDI (**B**), or the PDI fragments, **abb’** (**C**), **abb’x** (**D**), **bb’xa’ **(**E**), **bb’xa’c **(**F**), **b’xa’c** (**G**), **a** (**H**), **ab** (**I**), or **a’c** (**J**) by insulin reduction assay. Rutin was used as a positive control. **K**. Quantitative statistical analyses of relative reductase activity as affected by TA and rutin. *n* = 3. Data are means ± SEM. A two-way ANOVA test was used, **p < 0.01, ***p < 0.001. n = the number of biological replicates. ns = no significant difference. DMSO: Dimethyl sulfoxide

Because TA has been reported as a potent PDI inhibitor [29], we further characterized the molecular basis for the interaction between them. To investigate the binding site of TA on the PDI protein, we conducted insulin reduction assays to evaluate its inhibitory effect on full-length PDI (**abb’xa’c**) as well as on various PDI fragments (the **a**, **ab**, **abb’**, **abb’x**, **bb’xa’**, **bb’xa’c**, **b’xa’c**, and** a’c** domains). Rutin is a well-characterized inhibitor of PDI due to its binding to the b’ domain and was used as a positive control. In the insulin reduction assay, TA effectively inhibited the reductase activity of PDI in a dose-dependent manner. The IC_50_ of TA inhibition of PDI was determined to be 0.82 µM (Fig. 1A). At this concentration, TA effectively inhibited the reductase activity of full-length PDI as well as the **b’**-containing fragments, **abb’**, **abb’x**, **bb’xa’**, **bb’xa’c**, and **b’xa’c** (Figs. 1B–1G). Conversely, TA showed minimal inhibitory effect on the activities of the three **b’**-free fragments, **a**, **ab**, and **a’ c** (Fig. 1H and K). Based on these results, we speculated that TA inhibits PDI by binding to the **b’** domain.

### TA binds non-covalently to the PDI protein and causes conformational changes in it

**Fig. 2:**
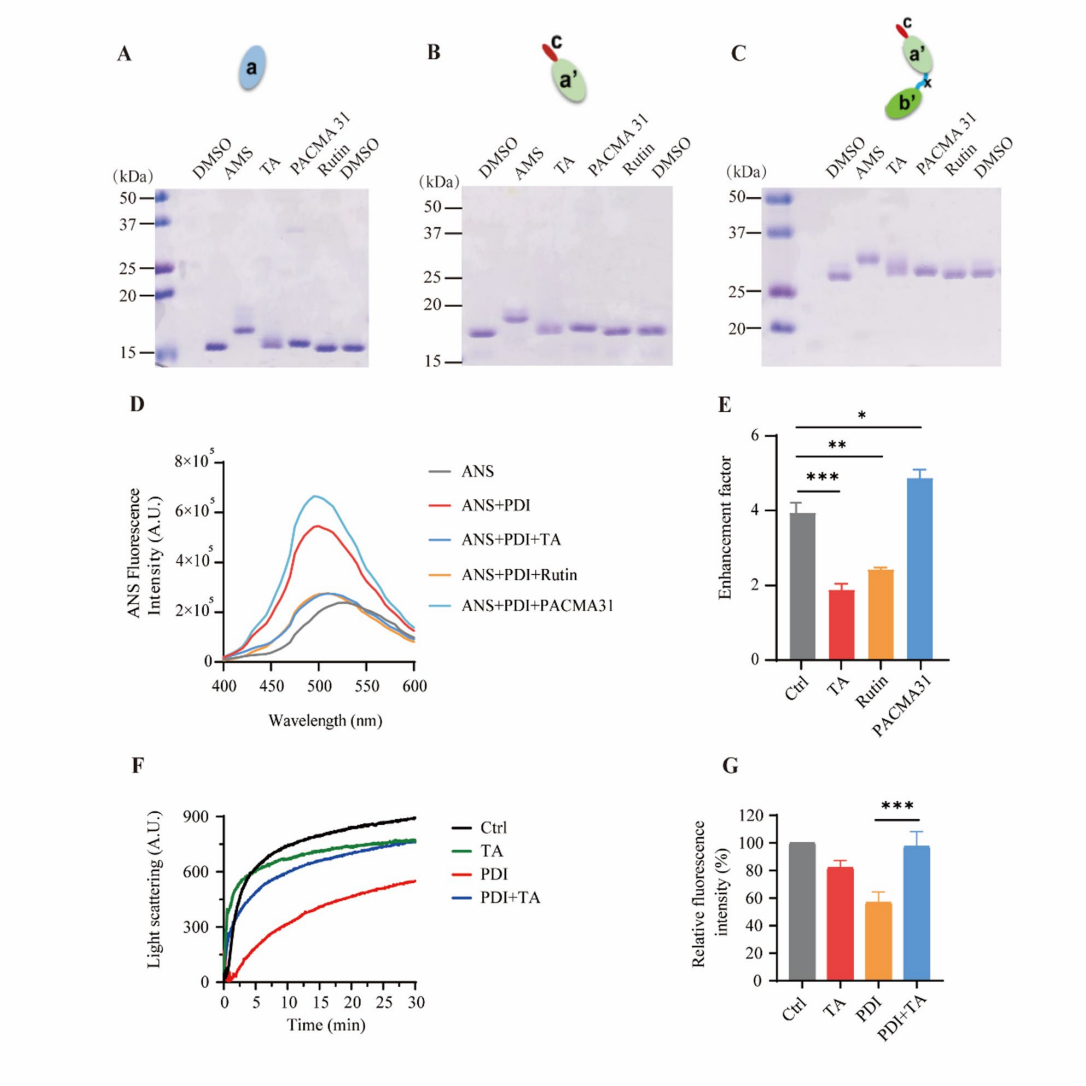
Tannic acid (TA) binds non-covalently to the protein disulfide isomerase (PDI), causing conformational changes. **A–C**. Detection of the binding mode between TA and the PDI fragments **a** (**A**), **a’c** (**B**) and **b’xa’c** (**C**) using non-reducing SDS-PAGE. AMS: positive control. The full-range molecular weight markers (kDa) are indicated on the left. The upward shift in electrophoretic mobility after AMS modification, indicative of the covalent binding to free sulfhydryl groups, is clearly demonstrated. **D**. Detecting the effect of TA on the PDI protein conformational changes using ANS dye. PACMA 31, a positive control, increases the hydrophobic exposure of PDI. **E**. Quantitative statistics of the enhancement factor in the binding of ANS to the PDI protein. *n* = 3. Data are means ± SEM. A one-way ANOVA test was used, *p < 0.05, **p < 0.01, ***p < 0.001. *n* = the number of biological replicates. AMS: 4-acetamido-4′-maleimidylstilbene-2,2′-disulfonate; ANS: 8-anilinonaphthalene-1-sulfonic acid; DMSO: dimethyl sulfoxide

Next, we explored the mechanism by which TA inhibited the enzyme activity of PDI. 4-acetamido-4′-maleimidylstilbene-2,2′-disulfonate (AMS) is a sulfhydryl modifier, and its modification of the free sulfhydryl groups (-SH) in proteins results in a corresponding increase in molecular weight, resulting in slower electrophoretic mobility in SDS-PAGE analysis [33]. Using AMS as a positive control, it was possible to detect whether the PDI small molecule inhibitor binds covalently to PDI using non-reducing SDS-PAGE analysis. Because both the **a** and **a’** domains contain a -CGHC- active site, responsible for thiol-disulfide interchange reactions, the PDI fragments **a**, **a’c**, and **b’xa’c** subjected to AMS modification showed slower SDS-PAGE electrophoretic mobility (Fig. 2A and C). On the contrary, TA and rutin showed very little effect on the SDS-PAGE migration rate (Fig. 2A and C), indicating that these two compounds do not bind covalently to the -SH of PDI. In addition, PACMA 31, an irreversible PDI inhibitor that forms a covalent bond with cysteine at the active site of PDI [34], slightly slowed the migration of the PDI fragments (Fig. 2A and C). 8-Anilinonaphthalene-1-sulfonic acid (ANS) is a hydrophobic fluorescent probe that binds to the hydrophobic exposure site of proteins [35]. In this study, ANS was used to investigate the conformational and hydrophobic changes in the PDI protein caused by TA binding. The results showed that TA and rutin decreased the binding of ANS to the PDI protein, while PACMA 31 enhanced the binding of ANS to the PDI protein (Fig. 2D and E), suggesting that TA and rutin binding led to conformational changes and a reduction in the hydrophobic exposure of PDI. Meanwhile, PDI-mediated refolding of denatured rhodanese protein was reduced in the presence of TA, indicating that the molecular chaperone activity of PDI had been inhibited (Fig. 2F and G). In summary, TA binds non-covalently to the PDI protein and induces conformational changes in it.

### TA effectively inhibits TNF-α signaling in cells

**Fig. 3:**
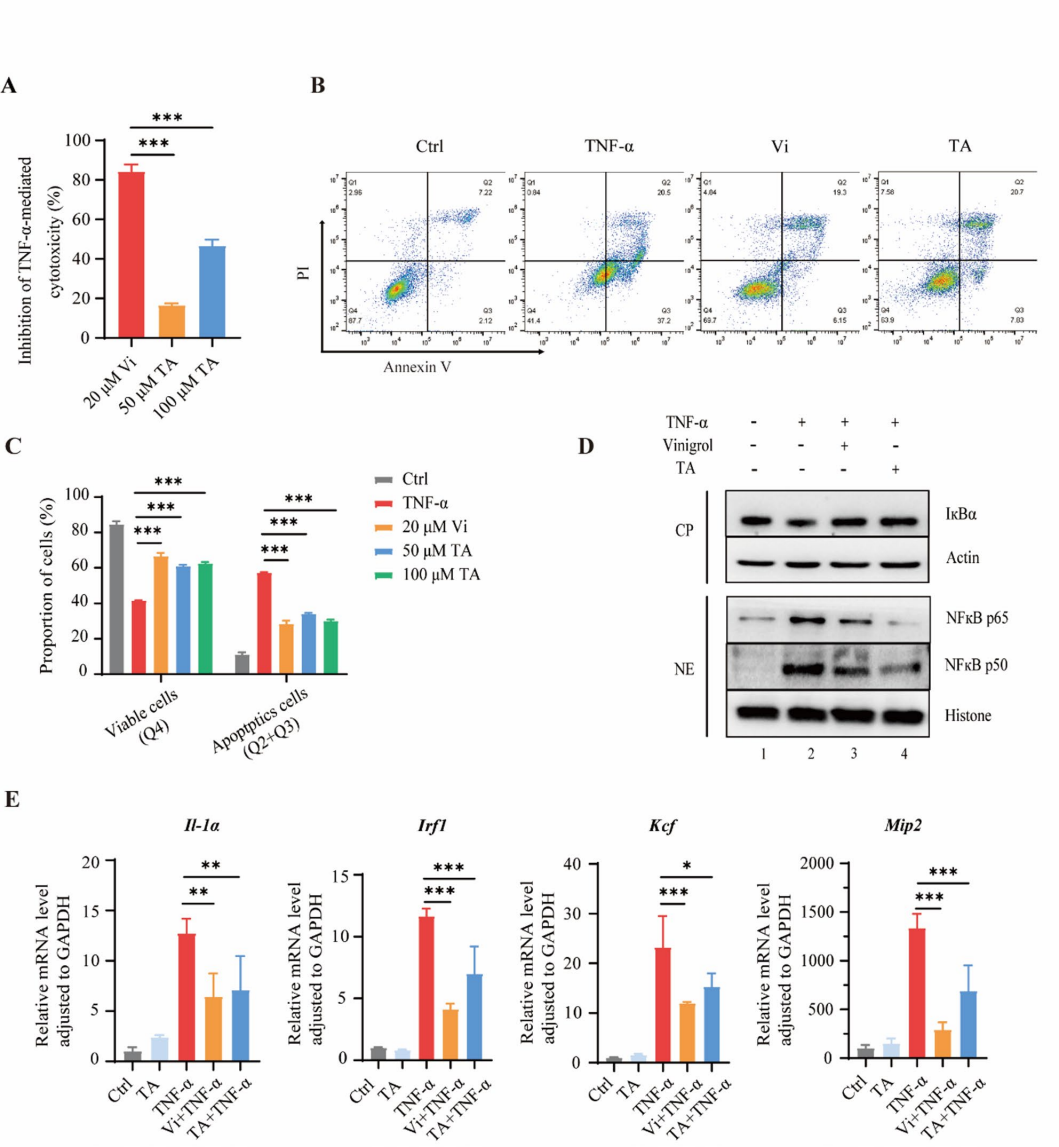
Tannic acid (TA) effectively inhibits TNF-α signaling. **A**. TA dependently inhibits TNF-α-induced L929 cell death, as revealed by MTS assay. Vinigrol (Vi), exerts a positive control to decrease TNF-α-induced cell death. L929 cells treated with 20 µM Vi and 50 or 100 µM TA for 8 h. **B**. Vi or TA reduces TNF-α-induced L929 cell apoptosis, as revealed by flow cytometry. Control (Ctrl: cells treated with TNF-α for 8 h), Vi (cells treated with TNF-α and 20 µM Vi for 8 h), TA (cells treated with TNF-α and 100 µM TA for 8 h). **C.** Quantitative statistics of L929 cell apoptosis, as revealed by flow cytometry. L929 cells treated with dimethyl sulfoxide (DMSO, TNF-α, TNF-α + 20 µM Vi, 50 µM TA + TNF-α, and 100 µM TA + TNF-α for 8 h. **D**. TA treatment inhibits the degradation of IκBα in the cytoplasm (CP) and activation of the NF-кB pathway in the nucleus (NE) induced by TNF-α. **E**. TA treatment inhibits the expression of NF-кB-targeted genes, including I*l-1α*, *Irf1*, *Kcf* and *Mip2*. Cells treated with 100 µM TA or 20 µM Vi for 4 h, then with added TNF-α for 0.5 h. *n* = 3. Data are means ± SEM. A one-way ANOVA and two-way ANOVA tests were used, *p < 0.05, **p < 0.01, ***p < 0.001. *n* = the number of biological replicates

Our previous study successfully demonstrated that PDI plays a positive role in TNF-α signaling, while vinigrol effectively blocks TNF-α signaling by inhibiting the PDI protein on the cell membrane [22]. Here, we examined whether TA could inhibit TNF-α signaling by targeting the PDI protein at the cellular level. Firstly, we tested the cytotoxicity of TA against L929 cells to ensure its safe dosage. The results showed that TA’s cytotoxicity against L929 cells was insignificant even at very high concentrations (Fig. S1B). 20 µM vinigrol showed a potent inhibitory effect against TNF-α-induced L929 cell death, while TA at a concentration of 100 µM exhibited significant antagonistic activity (Fig. 3A). In order to further investigate how TA inhibits TNF-α-induced cytotoxicity, we explored whether TA could inhibit cell apoptosis. Flow cytometry results showed that TNF-α treatment induced apoptosis in L929 cells, while treatment with vinigrol or TA inhibited apoptosis (Fig. 3B). TNF-α treatment induced 57.2% apoptosis in L929 cells, while vinigrol intervention only resulted in 28.6% cell apoptosis. Surprisingly, the 50 µM and 100 µM TA treatments reduced the apoptosis percentage to 34.1% and 30.1%, respectively (Fig. 3C). Because TNF-α was able to activate the NF-кB-mediated inflammatory signaling pathway by inducing IкBα degradation and the release of NF-κB subunits, primarily p65 and p50 [11, 36], we tested whether TA could affect the NF-кB signaling pathway. We found that both vinigrol and TA treatment can inhibit the TNF-α induced degradation of IκBα in the cytoplasm and the activation of the NF-кB pathway in the nucleus (Fig. 3D). Correspondingly, the expression of NF-кB-targeted genes was elevated under TNF-α treatment, but inhibited under TA treatment (Fig. 3E). These results show that TA had an inhibitory effect on TNF-α signaling.

### TA induced-TNFR1 shedding depends on the PDI protein

**Fig. 4:**
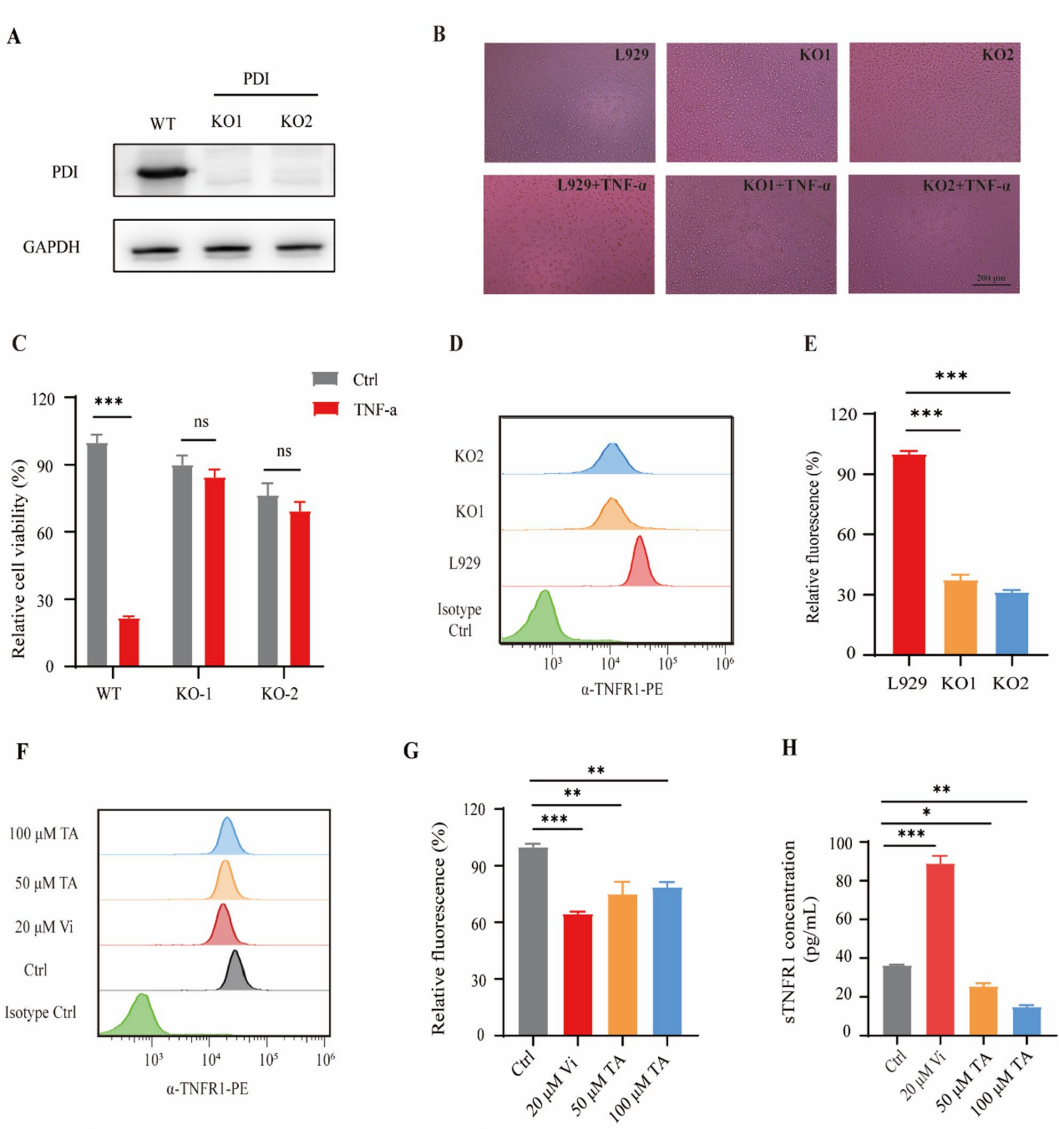
Tannic acid (TA) induced-TNFR1 shedding depends on the protein disulfide isomerase (PDI). **A**. Knockout effect of the PDI protein in L929 cells, as detected by western blotting. Knockout KO1 and KO2 are two different clones. **B.** L929 cells and PDI KO cells treated with TNF-α, as detected using a microscope. Cells treated with dimethyl sulfoxide (DMSO) (as the control) or TNF-α for 8 h. **C**. Activity of wild-type (WT) and PDI KO (KO-1 and KO-2) cells treated with TNF-α, as measured using MTS analysis. Cells treated with DMSO (as the control) or TNF-α for 8 h. **D.** The fluorescence intensity of TNFR1 on the membranes of L929 cells and PDI KO L929 cells, as detected by flow cytometry. **E.** Quantitative statistics of the TNFR1 fluorescence intensity on the cell membranes, as detected by flow cytometry. **F.** The fluorescence intensity of TNFR1 on the membranes of L929 cells treated with vinigrol (Vi) and TA for 8 h, as detected by flow cytometry. **G.** Quantitative statistics of the TNFR1 fluorescence intensity on cell membranes, as detected by flow cytometry. **H**. The change of soluble TNFR1 (sTNFR1) in the medium, as detected by ELISA. *n* = 3. Data are means ± SEM. A one-way ANOVA test was used, *p < 0.05, **p < 0.01, ***p < 0.001. *n* = the number of biological replicates. ns = no significant difference

As shown in our previous study, PDI can inactivate ADAM17, and inhibition of PDI by vinigrol can activate ADAM17 in cells to induce TNFR1 shedding [22]. To explore whether the inhibition of TNF-α signaling by TA is dependent on the PDI protein, we knocked out PDI in L929 cells using CRISPR-CAS9. Compared with the control cells, cells transfected with PDI CRISPR-CAS9 effectively reduced PDI protein levels (Fig. 4A). Consequently, due to the absence of the PDI protein, PDI KO cells exhibited a diminished response to TNF-α-induced cytotoxicity (Fig. 4B). The viability of PDI KO cells treated with TNF-α was almost unaffected compared with the control cells (Fig. 4C). After knocking out the PDI protein, the fluorescence intensity of TNFR1 on the membrane of PDI KO L929 cells decreased by more than 60% compared with normal L929 cells (Fig. 4D and E), further confirming the regulation of TNF-α signaling by PDI.

Next, we assessed the abundance of TNFR1 on L929 cell membranes following treatment with TA (Fig. 4F). Flow cytometry analysis showed that the fluorescence intensity of TNFR1 antibodies on the cell membrane was significantly reduced after TA treatment, similar to that after vinigrol treatment (Fig. 4G). Correspondingly, the soluble TNFR1 (sTNFR1) content in the supernatant after vinigrol treatment increased significantly, as measured by ELISA assay, consistent with the decrease in membrane TNFR1 (Fig. 4H). However, the measured sTNFR1 content following TA treatment showed an unexpected decrease, falling even below that of the control group, probably because polyphenols, such as TA, are highly adhesive natural compounds capable of forming conjugates with various macromolecules, particularly proteins [37]. Polyphenols can precipitate certain alkaloids, peptides, gelatin, and other proteins from solution via hydrogen bonding and hydrophobic and ionic interactions [38]. Therefore, TA may precipitate with sTNFR1 in the supernatant, leading to decreased sTNFR1 content. Altogether, our findings indicate that TA, like vinigrol, can inhibit the PDI protein, leading to TNFR1 shedding and subsequently increasing the inhibitory effect of TNF-α.

### TA alleviates symptoms in an IMQ-induced psoriasis mouse model

**Fig. 5:**
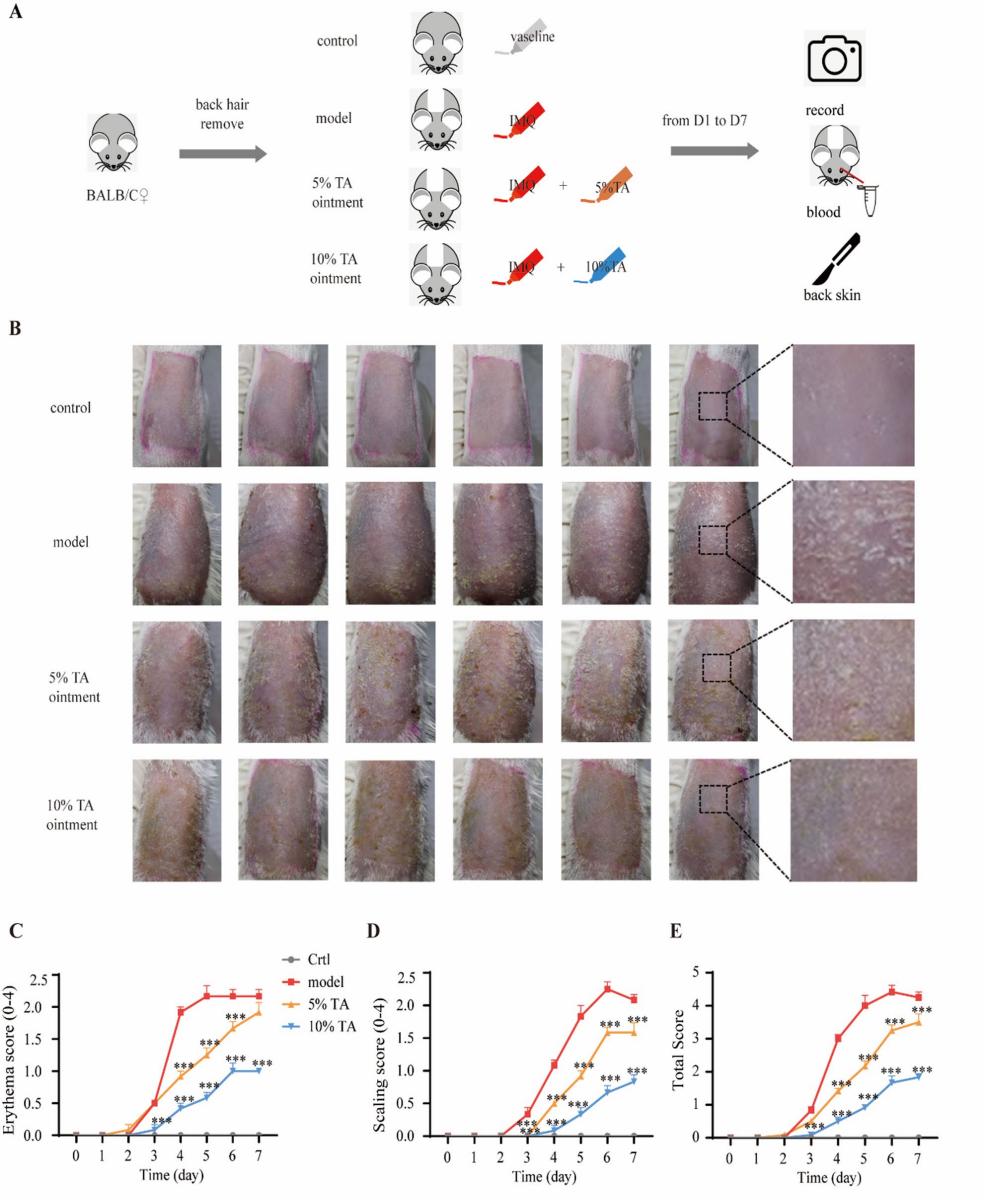
Tannic acid (TA) alleviates symptoms in an imiquimod-induced psoriasis mouse model. **A**. The effect of TA on psoriasis in a mouse model induced by imiquimod (IMQ). The back hair was shaved from all of the mice to enable application of the drug onto the skin. IMQ is a classic psoriasis model that induces inflamed squamous skin lesions similar to plaque psoriasis. Control: the mice were treated with Vaseline in the morning and afternoon. Model: the mice were treated with IMQ in the morning and Vaseline in the afternoon. 5% TA and 10% TA: the mice were treated with IMQ in the morning, and 5% TA or 10% TA ointment in the afternoon. All treatments lasted one week. **B**. The back skin of the mice is shown on each day. **C–E**. The rash area (**C**), scurf area (**D**), psoriasis severity index (PASI) (**E**) scores of all mice were recorded from day 1 to day 7. *n* = 6. Data are means ± SEM. A two-way ANOVA test was used, ***p < 0.001. *n* = the number of biological replicates

**Fig. 6:**
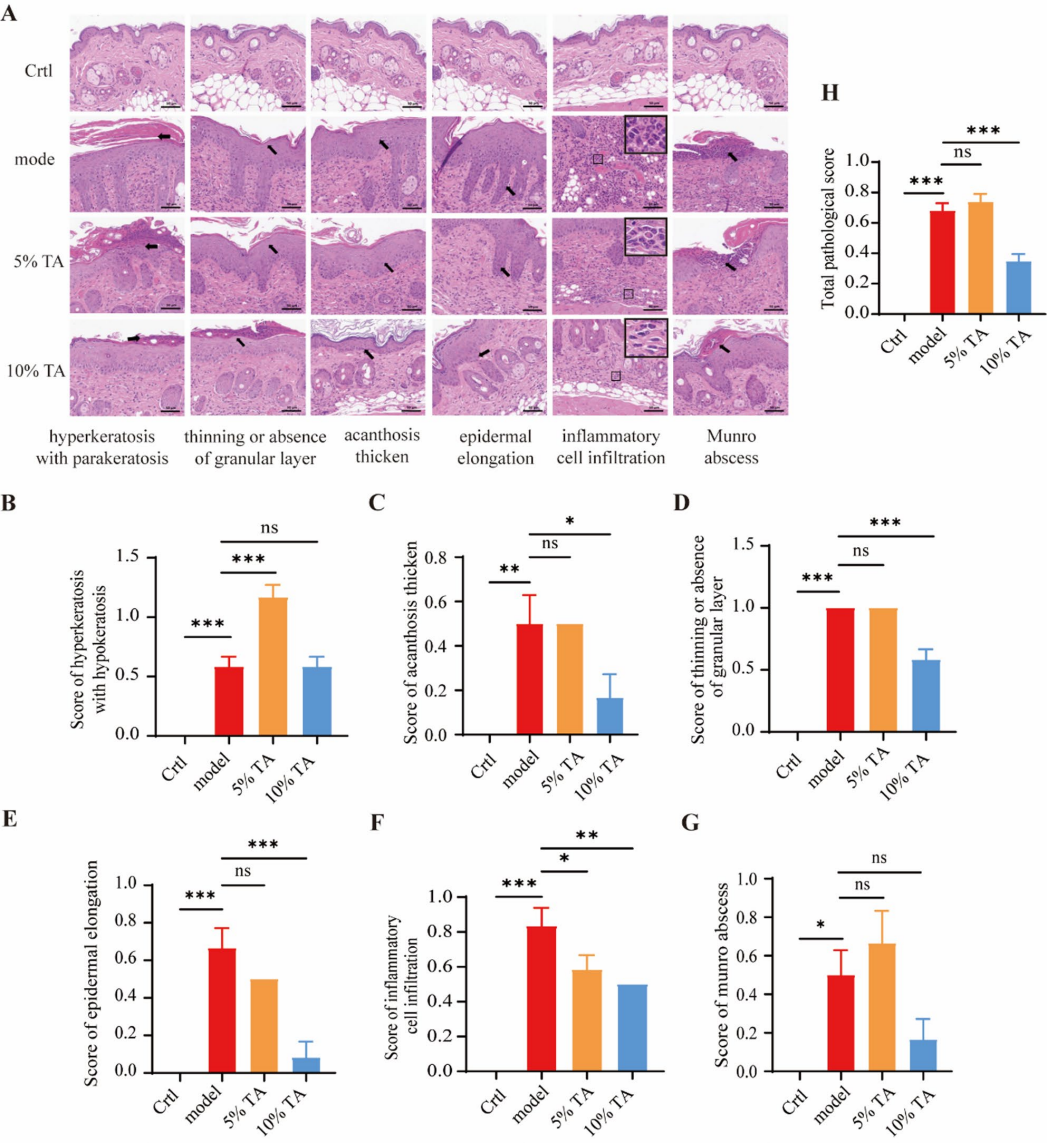
Tannic acid (TA) alleviates the pathological symptoms in an imiquimod-induced psoriasis mouse model. **A**. The typical psoriasis pathological features of the back tissue of mice, shown by hematoxylin and eosin (HE) staining. **B–G** Quantitative statistics of the histopathological scores: hyperkeratosis with parakeratosis (**B**); thinning or absence of the granular layer (**C**); acanthosis thickening (**D**); epidermal elongation (**E**); inflammatory cell infiltration; (**F**); Munro abscess occurrence (**G**); and the total pathological score of the six individual HE histopathologic scores **H**. *n* = 6. Data are means ± SEM. A one-way ANOVA test was used, *p < 0.05, **p < 0.01, ***p < 0.001. *n* = the number of biological replicates. ns = no significant difference

TA effectively induced the shedding of TNFR1 in vitro, suggesting its potential application in the treatment of psoriasis. Therefore, we explored the effect of TA treatment in a widely used IMQ-induced psoriasis mouse model [39]. IMQ was applied to the back skin of the mice once a day in the morning, and the corresponding vehicle ointment and different concentrations of TA ointment were applied in the afternoon (Fig. 5A). The skin rash and scurf on the backs of the mice were recorded and evaluated every day from day 1 to day 7, and the degree of psoriasis was determined using the psoriasis area and severity index (PASI) score. The model group exhibited more skin damage, and larger rash and scurf areas compared with the control group (Fig. 5B), as evidenced by elevated rash scores (Fig. 5C), scurf scores (Fig. 5D), and PASI scores (Fig. 5E). The intervention groups treated with different dosages of TA showed varying degrees of rash and scurf compared with the model group (Fig. 5B). The 10% TA intervention group showed significant reductions in both rash and scurf, as evidenced by reduced PASI scores (Figs. 5C–E). To further analyze the beneficial effects of the drug treatments, pathological sections of mouse back skin were evaluated. Hematoxylin and eosin staining showed that the skin tissue of the control group was normal with evenly arranged layers (Fig. 6A). In contrast, the model group showed hyperkeratosis with parakeratosis (Fig. 6B), mild acanthosis thickening (Fig. 6C), mild segmental thinning or absence of a granular layer (Fig. 6D), mild elongation of the epidermal processes (Fig. 6E), diffuse infiltration of mixed inflammatory cells (Fig. 6F), and the mild occurrence of focal Munro abscesses (Fig. 6G). However, the TA ointment intervention groups with different dosages showed varying degrees of improvement in skin pathology regarding these aspects (Fig. 6A). Notably, the 10% TA ointment treatment group exhibited significantly greater improvements (Fig. 6B and 6G). The HE total pathological score of the model group was higher than that of the control group, while treatment with 10% TA ointment reduced the pathological score and mitigated the severity of psoriasis in the mice (Fig. 6H).

### TA treatment restores balance to physical functions and blood inflammation markers in the psoriasis mouse model

**Fig. 7:**
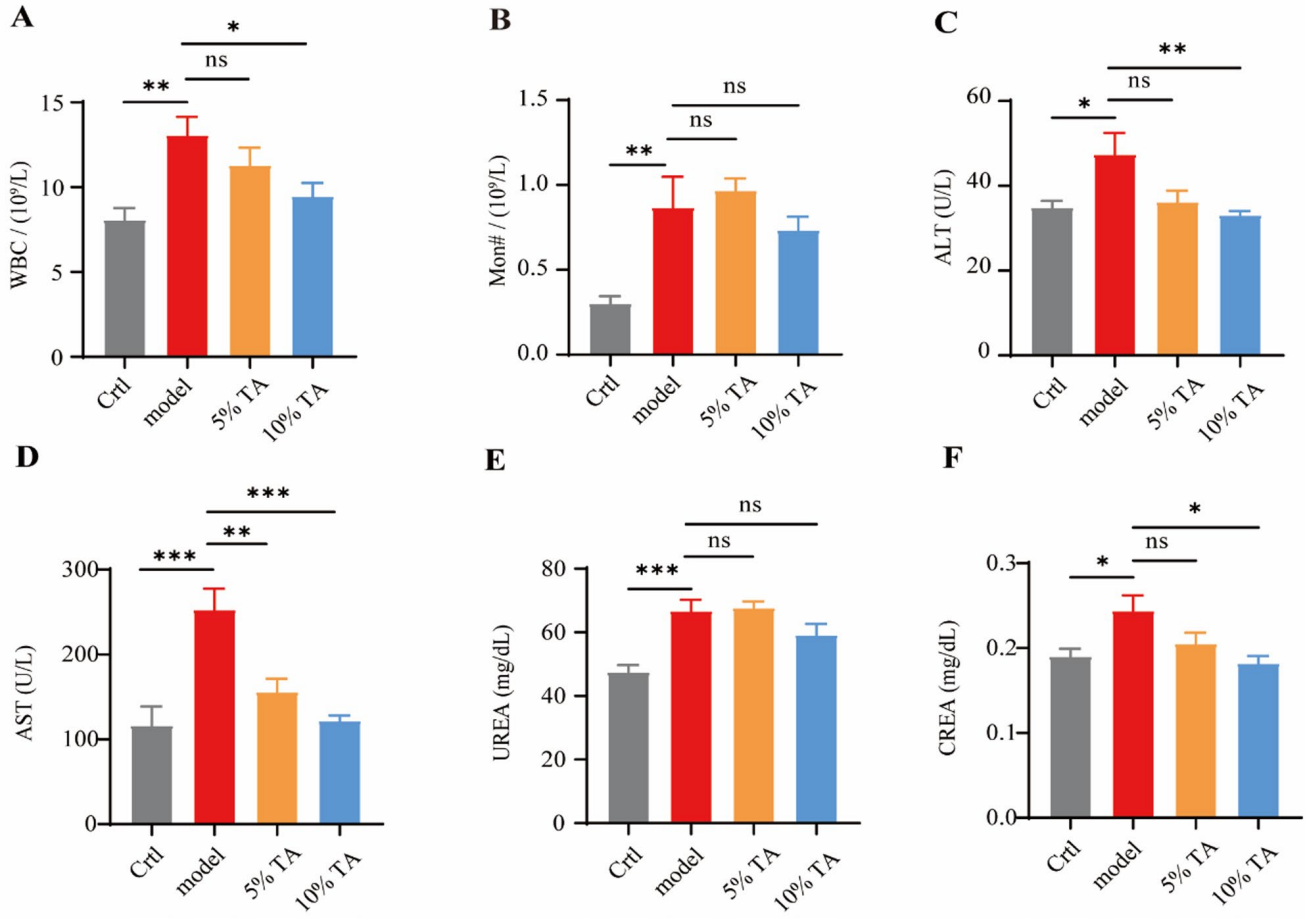
Tannic Acid (TA) treatment restores balance to physical functions and blood inflammation markers in a psoriasis mouse model. A and B. The number of white blood cells (**A**) and monocytes (**B**) as detected by routine blood examination. **C–F**. alanine aminotransferase (ALT) (**C**), aspartate aminotransferase (AST) (**D**), urea (UREA) (**E**), and creatinine (CREA) (**F**) in the mouse blood as measured by blood biochemical testing. *n* = 6. Data are means ± SEM. A one-way ANOVA test was used, *p < 0.05, **p < 0.01, ***p < 0.001. *n* = the number of biological replicates. ns = no significant difference

Because we demonstrated that TA ointment could mitigate psoriasis in vivo, we further analyzed its potential effects on inflammation and physiological function to ensure its safety. Compared with the control group, the IMQ model group showed a rapid increase in the number of white blood cells (Fig. 7A), monocytes (Fig. 7B), and neutrophils (Fig. S1C), indicating the activation of inflammatory responses in the circulatory system. Treatment with TA ointment significantly reduced the levels of these inflammatory cells. Additionally, IMQ treatment led to a decrease in the lymphocyte and red blood cell counts in the blood of the mice (Fig. S1D and S1E), while TA ointment treatment partially restored their levels. Notably, our study also demonstrated that TA ointment effectively improved liver and kidney function in the psoriasis mice. Compared with the control group, the IMQ model group showed significant increases in alanine aminotransferase (ALT) (Fig. 7C), aspartate aminotransferase (AST) (Fig. 7D), urea (UREA) (Fig. 7E), and creatinine (CREA) (Fig. 7F) levels, whereas treatment with TA significantly reduced these levels, even bringing them back to the normal ranges. In conclusion, these results indicate that TA ointment treatment effectively restored the physiological functions of psoriasis mice to a balanced status.

## Discussion

Natural compounds, derived from a wide range of sources at low cost, have been present throughout evolutionary history and have lower toxicity compared to man-made drugs. This study set out to investigate the inhibitory effect of the naturally occurring compound TA on TNF-α signaling, and its potential application in the treatment of autoimmune diseases such as psoriasis. Initially, we confirmed that PDI is a direct protein target for TA. Analysis of the molecular mechanism of TA action, showed that noncovalent binding between TA and the PDI protein leads to structural changes that induce TNFR1 shedding, thereby impeding TNF-α signaling and so counteracting its cytotoxic effects. Furthermore, we confirmed that TA effectively alleviates psoriasis symptoms in a mouse model. This study highlights the potential for TA as an effective therapeutic drug for autoimmune diseases.

Since TNF-α plays a pivotal role in the pathological mechanism of psoriasis [40], targeting TNF-α signaling to disrupt the inflammatory cycle of psoriasis can enhance therapeutic efficacy and benefit patients. Notably, our previous study showed that regulating the PDI-ADAM17-TNFR1 signal module using small molecules could inhibit TNF-α signaling at the cellular level and alleviate autoimmune disease in a mouse model [22]. Because the naturally derived polyphenol TA had been reported as a potent PDI inhibitor [29], we hypothesized that TA would also show promise in inhibiting TNF-α signaling and psoriasis. This study provides biochemical, cellular, and in vivo evidence to confirm this hypothesis. First, we demonstrated that TA binds non-covalently to the **b’** domain of the PDI protein, causing conformational changes (Fig. 2A-2E). Second, we found that TA-induced-TNFR1 shedding depends on the PDI protein (Fig. 4D-4H), resulting in effective inhibition of TNF-α signaling in L929 cells (Fig. 3A-3E). More importantly, 10% TA ointment treatment significantly mitigated the severity of psoriasis (Fig. 5B-5E) and reduced its pathological scores (Fig. 6A-6H) in an IMQ-induced psoriasis mouse model. We also observed that TA is a potent PDI inhibitor in biochemical assays (Figs. 1K and 2E), but is less effective than vinigrol in inhibiting TNF-α-mediated toxicity (Fig. 3A) and TNFR1 shedding (Fig. 4G) in cellular assays. A potential explanation for these conflicting results is that these two compounds bind to different sites in the **b’** domain of PDI, leading to varying degrees of conformational change in the PDI protein. This could result in distinct regulatory effects on PDI-mediated inhibition of ADAM17 under the physiological conditions previously reported [22]. Overall, this study not only demonstrated the potential for TA in relieving psoriasis but also validated PDI as a reliable target for alleviating autoimmune diseases through regulating TNF-α signaling.

Developing efficient and safe pharmaceutical treatments for psoriasis presents challenges. Established systemic drugs, such as ciclosporin, methotrexate, and acitretin, have limited effectiveness and can cause severe liver and kidney toxicity and even teratogenicity [41]. On the other hand, monoclonal antibodies against TNF-α, such as infliximab, adalimumab, and etanercept cost 10 times more than traditional systemic drugs [41] and have potentially serious side effects such as triggering autoimmune antibody responses, suppressing human immune defenses, and increasing infection risk [42]. Therefore, easily extracted natural active substances with lower costs and fewer side effects are promising candidates to replace systemic drugs and monoclonal antibody TNF-α inhibitors. Besides its excellent efficacy in inhibiting TNF-α signaling and relieving psoriasis, TA has significant advantages in terms of safety. It has been recognized as a food additive by the Food and Drug Administration [43] and its tolerable dose is high according to previous studies. Its LD50 (50% mortality rate) is 2260 mg/kg in rats, and its daily tolerable intake in humans is 13.6 g/60 kg body weight [44]. No abnormalities were found in the exercise habits, body weight, feeding behavior, or organ morphology of mice after administering 30 mg/kg/d TA for six months [45]. Consistent with these reports, the safety of TA was also demonstrated in our study. The number of inflammation-related cells in the blood of mice increased after IMQ treatment [46], whereas TA treatment could restore these levels to almost normal (Fig. 7A and 7B). Additionally, while the liver and kidney functions of the psoriasis model mice were compromised, key indicators of liver and kidney functions, such as ALT, AST, UREA and CREA, gradually returned to normal levels after TA treatment (Fig. 7C-7F). Overall, TA was shown to be an effective PDI inhibitor to block TNF-α signaling, offering an efficient, safe, and cost-effective option for treating autoimmune diseases such as psoriasis.

## Supplementary Information

Supplementary Material 1

Supplementary Material 2

## Data Availability

All data reported in this article will be shared upon reasonable request.

## Abbreviations

Act D: Actinomycin D
ALT: Alanine aminotransferase
AMS: 4-acetamido-4′-maleimidylstilbene-2,2′disulfonate
ANS: 8-Anilinonaphthalene-1-sulfonic acid
AST: Aspartate aminotransferase
CREA: Creatinine
DTT: Dithiothreitol
HE: Hematoxylin and eosin staining
IMQ: Imiquimod
KO: Knockout
OD: Optical density
PDI: Protein disulfide isomerase
PASI: Psoriasis area and severity index
-SH: Sulfhydryl groups
sTNFR1: Soluble TNFR1
TA: Tannic acid
TNF-α: Tumor necrosis factor alpha
