# Supplementary figures and images for: Tannic acid inhibits TNF-α signaling by targeting the protein disulfide isomerase and alleviates symptoms in an imiquimod-induced psoriasis mouse model
Source: Cell Commun Signal. 2025 Nov 29;24:46. doi: 10.1186/s12964-025-02535-y (PMC12837628; doi:10.1186/s12964-025-02535-y)

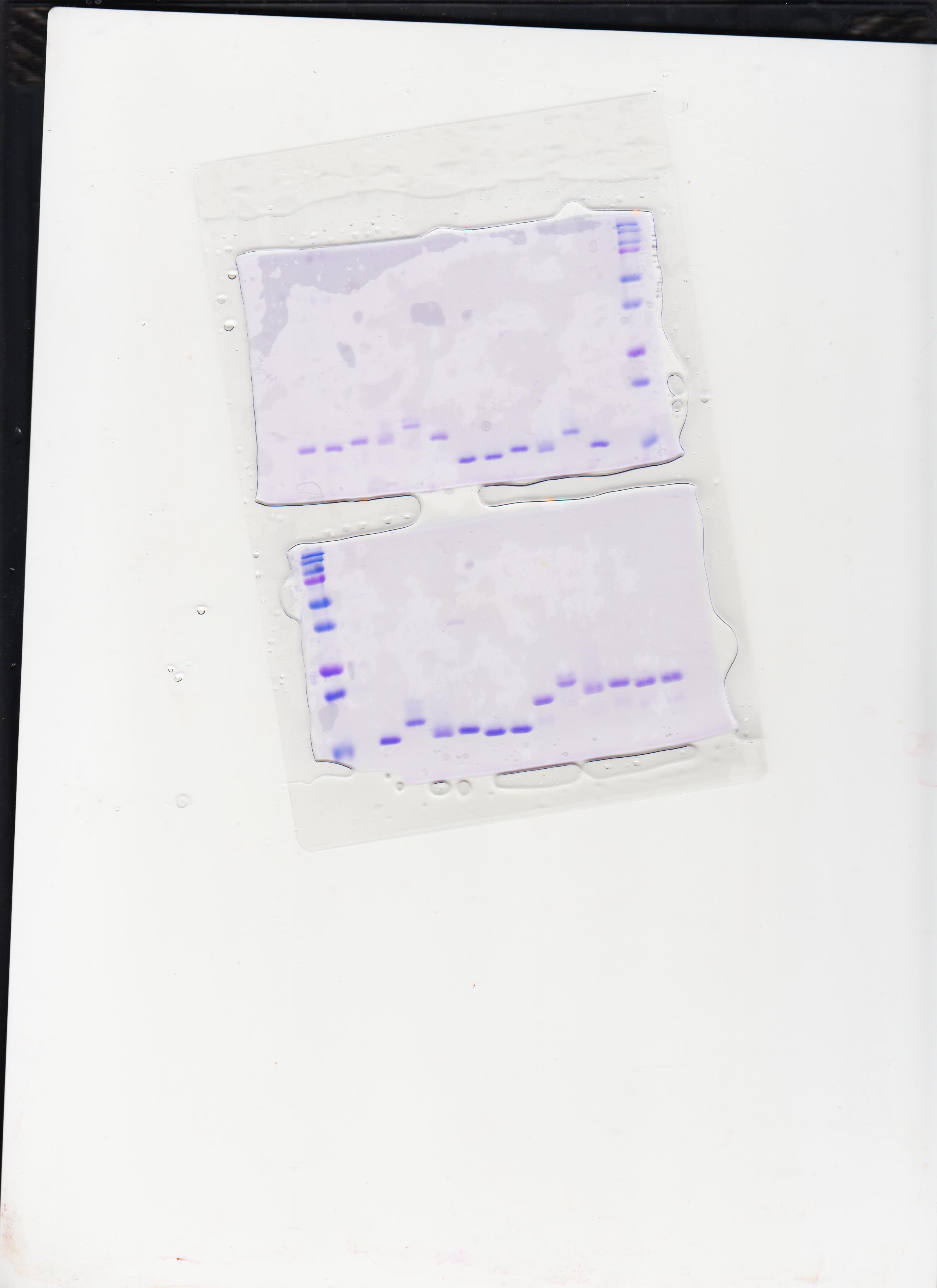

Supplement: Supplementary file 1 — Supplementary Material 1 [file 12964_2025_2535_MOESM1_ESM.zip › Gels and Blots images/Figure2A&2B.jpg]

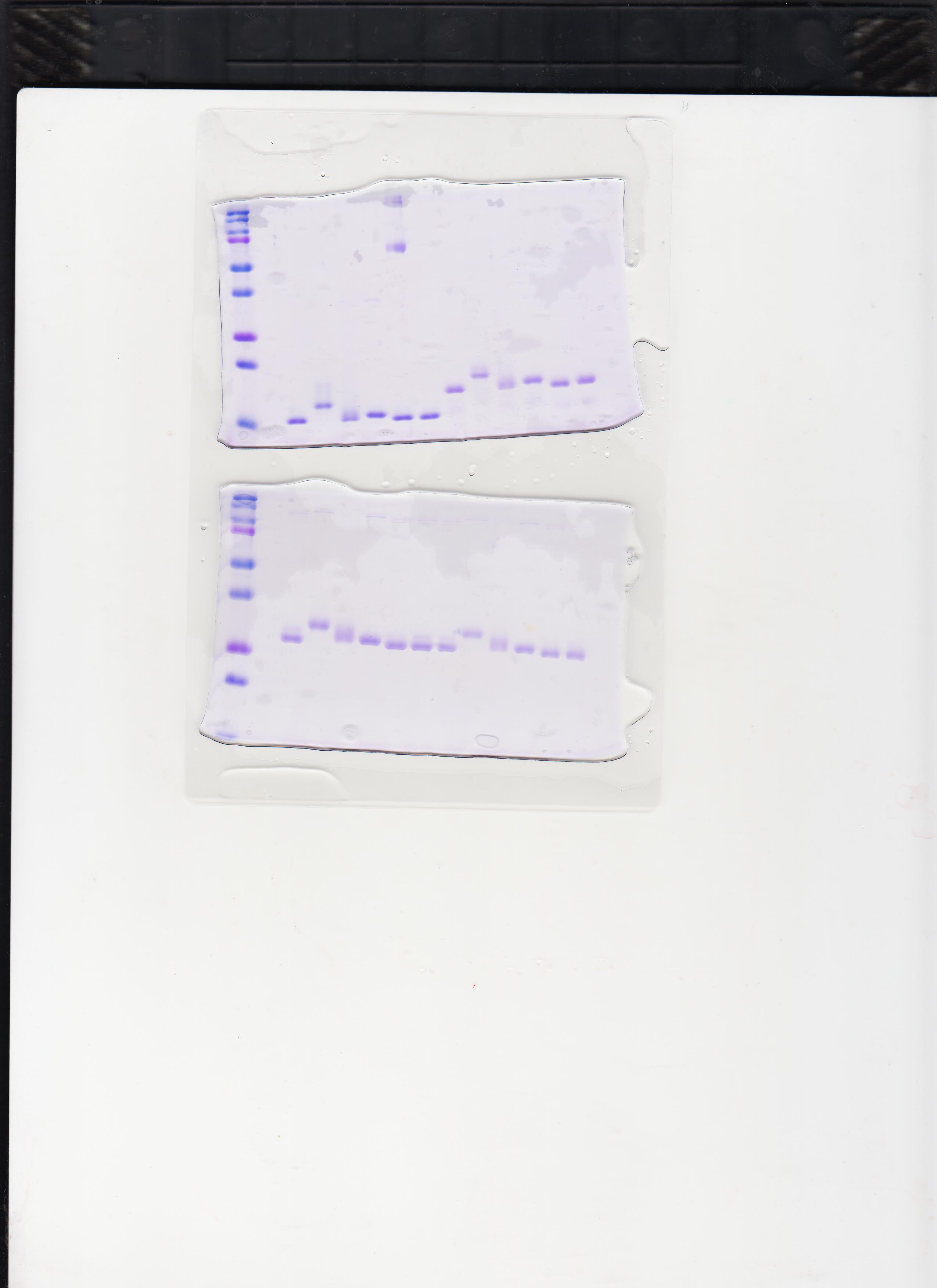

Supplement: Supplementary file 1 — Supplementary Material 1 [file 12964_2025_2535_MOESM1_ESM.zip › Gels and Blots images/Figure2C.jpg]

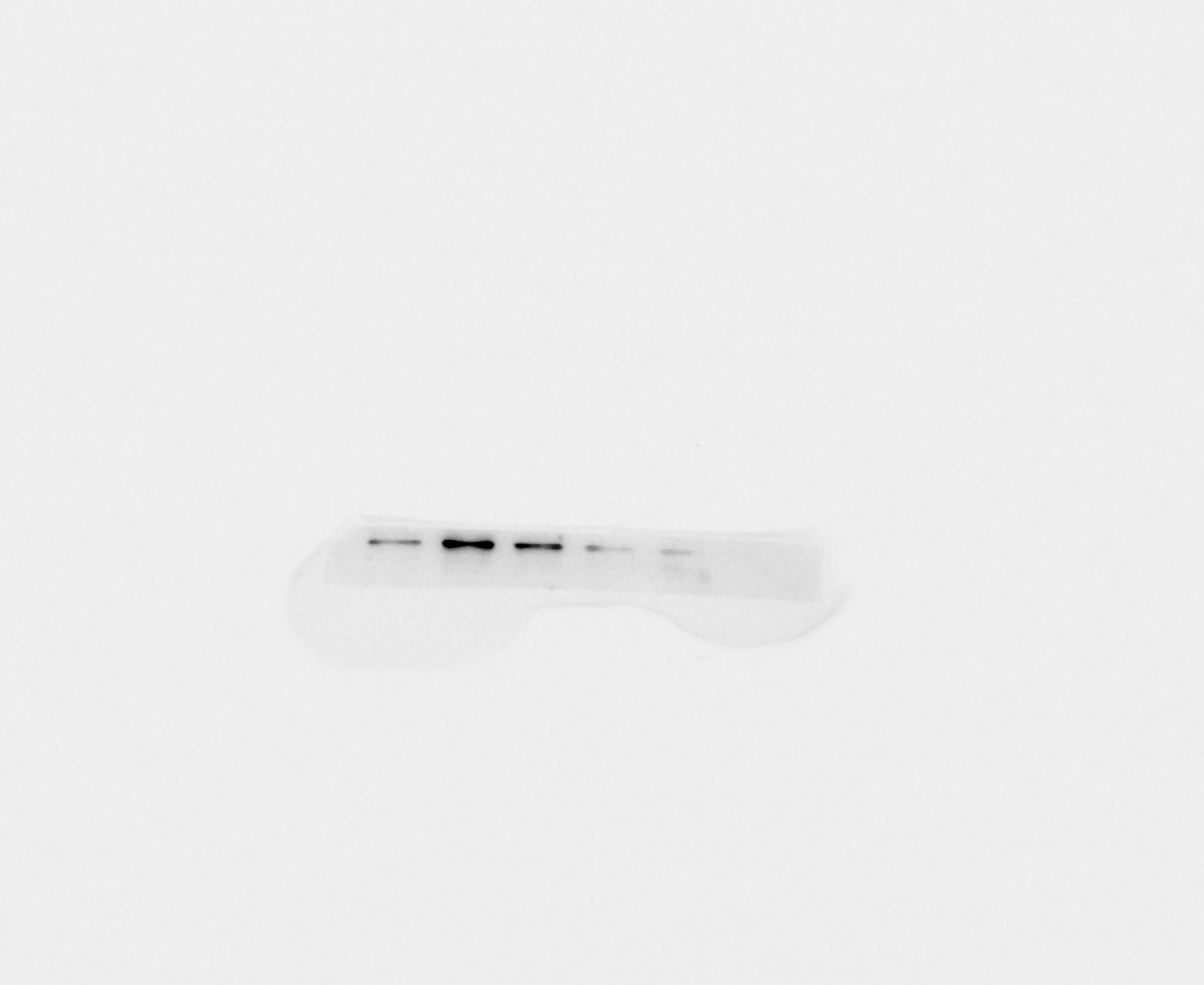

Supplement: Supplementary file 1 — Supplementary Material 1 [file 12964_2025_2535_MOESM1_ESM.zip › Gels and Blots images/Figure3D-1.tif]

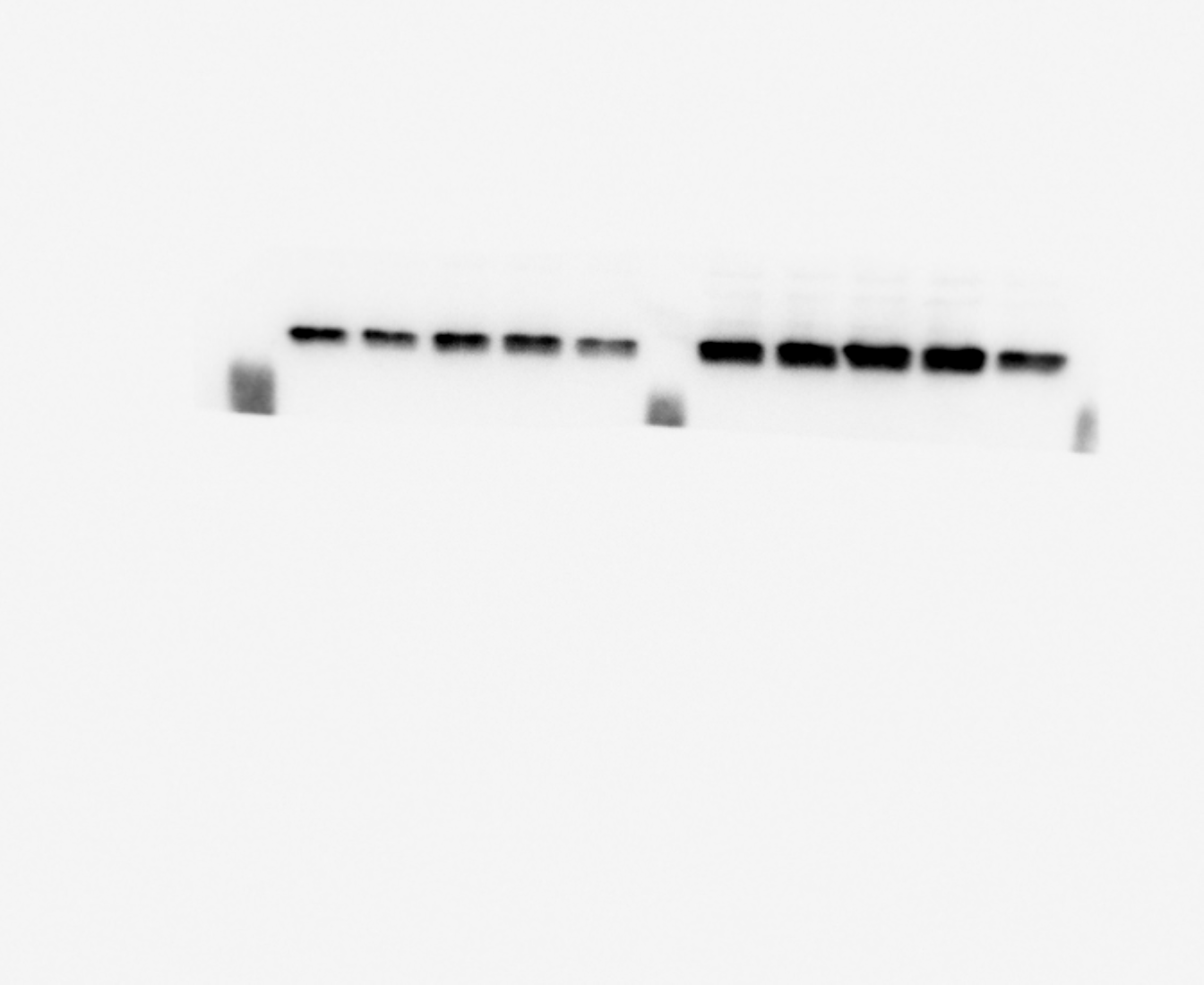

Supplement: Supplementary file 1 — Supplementary Material 1 [file 12964_2025_2535_MOESM1_ESM.zip › Gels and Blots images/Figure3D-2.tif]

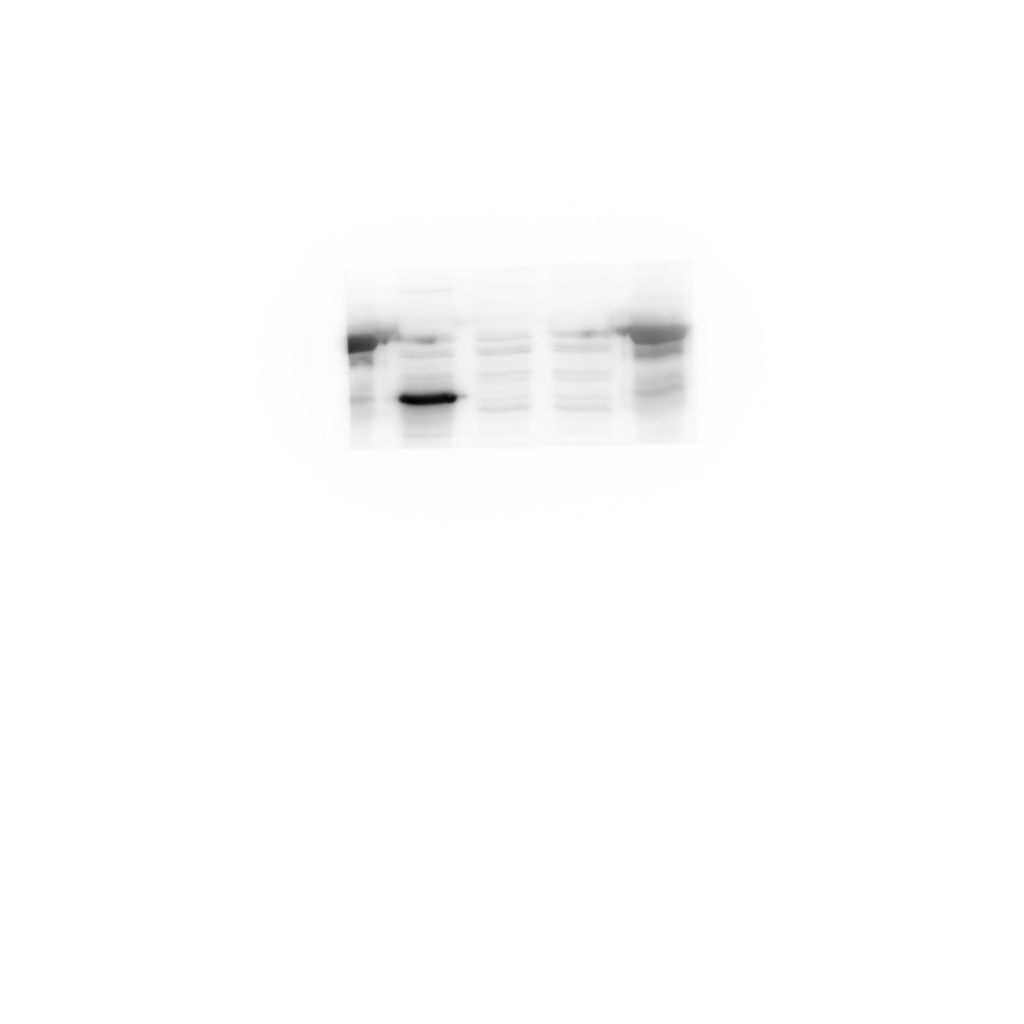

Supplement: Supplementary file 1 — Supplementary Material 1 [file 12964_2025_2535_MOESM1_ESM.zip › Gels and Blots images/Figure4A.jpg]
