# Supplementary material for: Tannic acid inhibits TNF-α signaling by targeting the protein disulfide isomerase and alleviates symptoms in an imiquimod-induced psoriasis mouse model
Source: Cell Commun Signal. 2025 Nov 29;24:46. doi: 10.1186/s12964-025-02535-y (PMC12837628; doi:10.1186/s12964-025-02535-y)
Supplement: Supplementary file 2 — Supplementary Material 2 [file 12964_2025_2535_MOESM2_ESM.docx]

**Supplemental information**

**Tannic Acid inhibits TNF-α signaling by targeting protein disulfide isomerase and alleviates symptoms in an imiquimod-induced psoriasis mouse model**

Wenhua Jin ^a, 1^, Yi Xia ^a, 1^, Shuo Sun ^b, c, 1^, Hejing Tang ^a^, Senyang Hu ^a^, Yan Zhang ^d^, Jiaqiang Huang ^a^, Ping Liu ^b, c^, Chenyun Hu ^b, c^, Jiayue Guo ^a^, Pengjie Wang ^a^, Peng An ^a^, Junjie Luo ^a^, Lei Wang ^b, c^, Fuqing Wang ^e, *^, Yongting Luo ^a,^ *****, Yinhua Zhu ^a,^ *****

^a^ Beijing Advanced Innovation Center for Food Nutrition and Human Health, Department of Nutrition and Health, China Agricultural University, Beijing, China, 100193

^b^ National Laboratory of Biomacromolecules, CAS Center for Excellence in Biomacromolecules, Institute of Biophysics, Chinese Academy of Sciences, Beijing, China, 100101

^c^ College of Life Sciences, University of Chinese Academy of Sciences, Beijing, China, 101408

^d^ College of Food Science and Engineering, Gansu Agricultural University, Lanzhou, China, 730070

^e^ Tibet Tianhong Science and Technology Co., Ltd, Xizang, China, 850000

***** Corresponding author

E-mail addresses: [fq7963@163.com (Q](mailto:fq7963@163.com%20(Q). Fu); luo.yongting@cau.edu.cn (Y. Luo); [zhuyinhua@can.edu.cn](mailto:zhuyinhua@can.edu.cn) (Y. Zhu)

^1^ These authors contributed equally to this paper


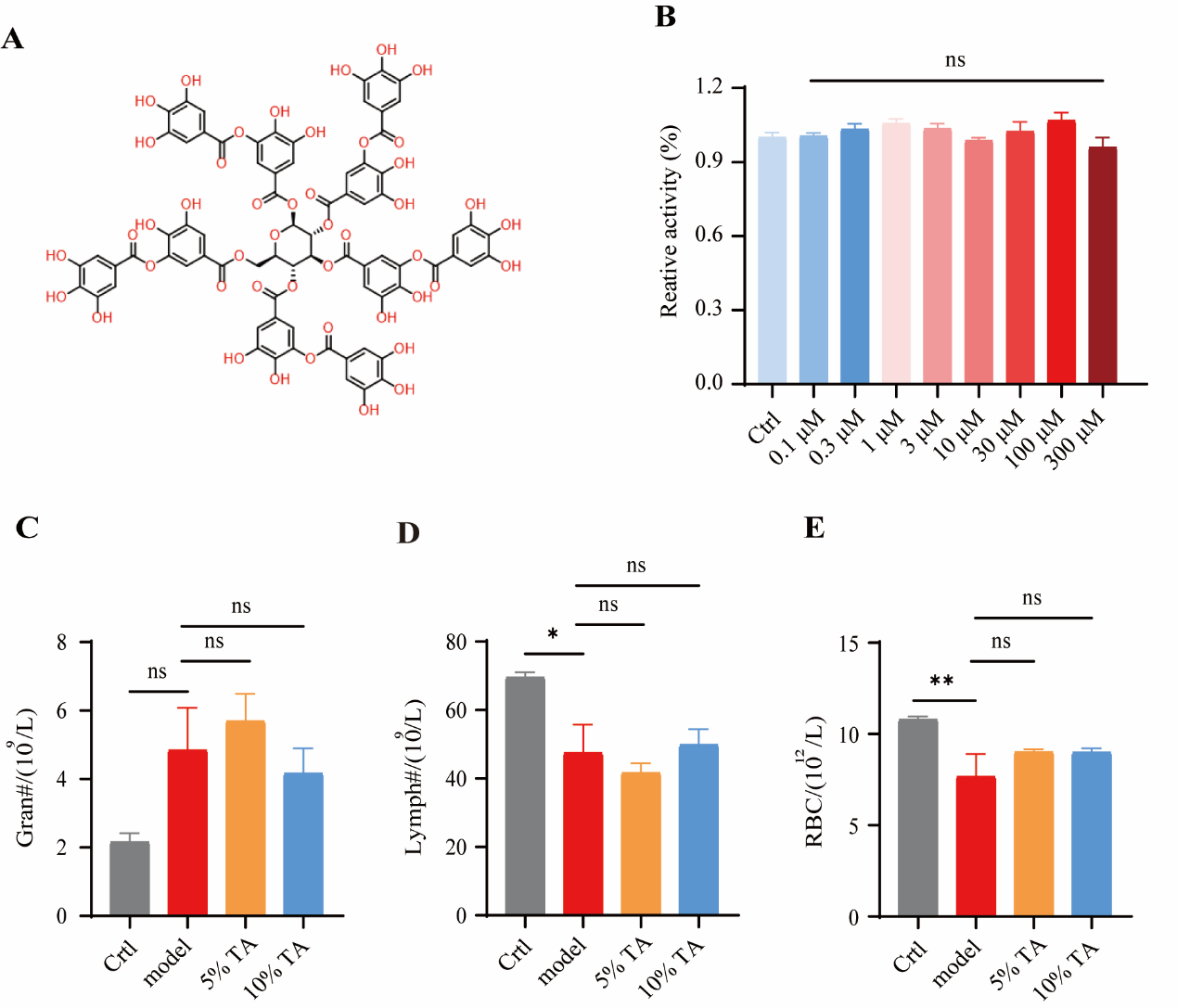


**Figure S1. A.** The molecular structure formula of TA. **B.** The cytotoxicity of TA to L929 cells is detected by MTS under different TA concentrations from 0.1 to 300 μM for 8 h. n=3. **C. D.E.** The number of Neutrophil (**C**), lymphocyte (**D**) and red blood cells (**E**) are detected by blood routine examination. n=6. Data are mean ± SEM. One-way ANOVA test is used, *p < 0.05, **p < 0.01. “n” represents the number of biological replicates. ns means no significance.
